# Transcriptional Mapping of the Human Cannabinoid Receptor 1 (*CNR1*) Gene Promoter

**DOI:** 10.3390/molecules31132387

**Published:** 2026-07-07

**Authors:** Alonso Cortez-Resendiz, Shivani S. Godbole, Nurgul Carkaci-Salli, Kent E. Vrana, Wesley M. Raup-Konsavage

**Affiliations:** 1 Department of Neuroscience and Experimental Therapeutics, Penn State College of Medicine, Penn State University, Hershey, PA 17033, USA; 2 Center for Cannabis and Natural Product Pharmaceutics (CCNPP), Penn State College of Medicine, Penn State University, Hershey, PA 17033, USA; 3 Department of Molecular and Precision Medicine, Penn State College of Medicine, Penn State University, Hershey, PA 17033, USA

**Keywords:** CNR1, cannabinoid receptor 1, cAMP, transcriptional regulation, promoter

## Abstract

The transcriptional regulation of the cannabinoid receptor 1 (CB1R) by promoter/enhancer elements and transcription factors is an area of cannabinoid research that has historically been understudied. To map the promoter region of the human *CNR1* gene (the gene encoding CB1R), a 997-base-pair fragment from the sequence upstream of the *CNR1* gene was cloned into a secreted luciferase reporter vector, and a series of deletion fragments were constructed. The transcriptional activity of these constructs was tested in human cell lines from three tissues: neuronal tissue (SHSY5Y), kidney tissue (HEK293T), and colonic epithelium (HCT116). Through this mapping, we have identified two key regulatory regions within the promoter. Increased levels of cAMP suppressed reporter expression from the full-length promoter fragment in all three cell lines, and in silico modeling predicts potential cAMP response elements (CRE) within one of the key regulatory sequences. Additionally, the minimal promoter region for *CNR1* also appears to be in the second regulatory region identified, and in silico modeling predicts BRE and INR elements within this sequence. These findings begin to unravel the mechanisms by which *CNR1* is transcriptionally regulated.

## 1. Introduction

The cannabinoid type 1 receptor (CB1R) is a G_i_/G_o_-coupled receptor activated by endocannabinoids (N-arachidonoylethanolamine [AEA] and 2-arachidonoylglycerol [2-AG]), phytocannabinoids (Δ^9^-tetrahydrocannabinol [(Δ^9^-THC]) as well as synthetic cannabinoids (WIN55212 and CP55940) and is primarily expressed in the central nervous system (CNS) [1,2,3,4,5,6]. Specifically, CB1R is highly expressed in the limbic system, cerebral cortex, and cerebellum [1,2,3,4,5,6]. The human cannabinoid type 1 receptor gene (*CNR1*) is located on chromosome 6q14-15, (the 91.8~96.1 cM locus) and lies within 15 cM of the D6S474 and D6S424 markers linked to schizophrenia [1,5,7]. The structure and transcriptional regulation of the *CNR1* gene, including its promoter, are currently not well understood and have received limited attention [1,2,3,4,5,7].

To date, research has focused on the activation/inhibition of the CB1R along with the upregulation/downregulation of this receptor in different human cell types, and disorders such as Alzheimer’s disease and schizophrenia. The overuse of cannabis can lead to the development of tolerance, which has been associated with decreased CB1R on the plasma membrane, that may ultimately lead to cannabis use disorder (CUD). Nevertheless, only a few publications have examined the transcriptional regulation of the *CNR1* gene promoter (located in the 5′ flanking region of the *CNR1* gene) responsible for the transcription of the CB1R protein [1,8]. While searching for the cause of these disorders and the regulation of the *CNR1* gene, investigators have examined changes in the *CNR1* promoter region, including an (AAT)*_n_* triplet repeat in the promoter region of the *CNR1* gene thought to be associated with the pathogenesis of schizophrenic disorders [7]. Studies involving luciferase reporter gene assays have shown transcriptional regulation in the *CNR1* gene [1,8]. First, the 1.0 kb 5′ flanking sequence of *CNR1* gene exon 1 showed significant promoter/enhancer-like activities [1]. Second, a single nucleotide polymorphism (SNP) found within the 3.0 kb immediately 5′ from the *CNR1* coding exon 3 showed reduced reporter gene expression when compared to the wild type [8]. More recently, the rs2180619 polymorphism (located about 3.0 kb from *CNR1* gene exon 1) and its related genotypes have been associated with attention deficits and reduction in working memory, behaviors like addiction and anxiety, as well as lowered levels of CB1R expression [1,9,10,11]. Finally, other studies have examined the role of transcriptional factors in the regulation of CB1R [4,12]. These findings clearly emphasize the need to explore the transcriptional regulation and structural composition of the *CNR1* gene and consequently the expression of the CB1R. An understanding of how the *CNR1* gene is regulated will contribute insights into human diseases and conditions.

To map the promoter regions of the human *CNR1* gene, a 997-bp fragment from the sequence upstream of the *CNR1* gene (similar to the 1.0 kb sequence theorized to contain the core promoter region in Zhang et al. [1]) was cloned into a secreted luciferase reporter construct (pNL2.3). A series of deletion fragments were constructed, and their activity was assessed in three different human cell lines. Through this mapping, we identified two key regions of transcriptional regulation. Additional data indicate that increases in cAMP levels can suppress *CNR1* gene expression in a cell-type specific manner. We report that the core promoter is located within the first 100 bp upstream of the *CNR1* gene sequence and predict several transcription factors that play a role in its regulation and implication in disease.

## 2. Results

### 2.1. Luciferase Activity of Full-Length CNR1 Promoter and Promoter Deletions

**Figure 1:**
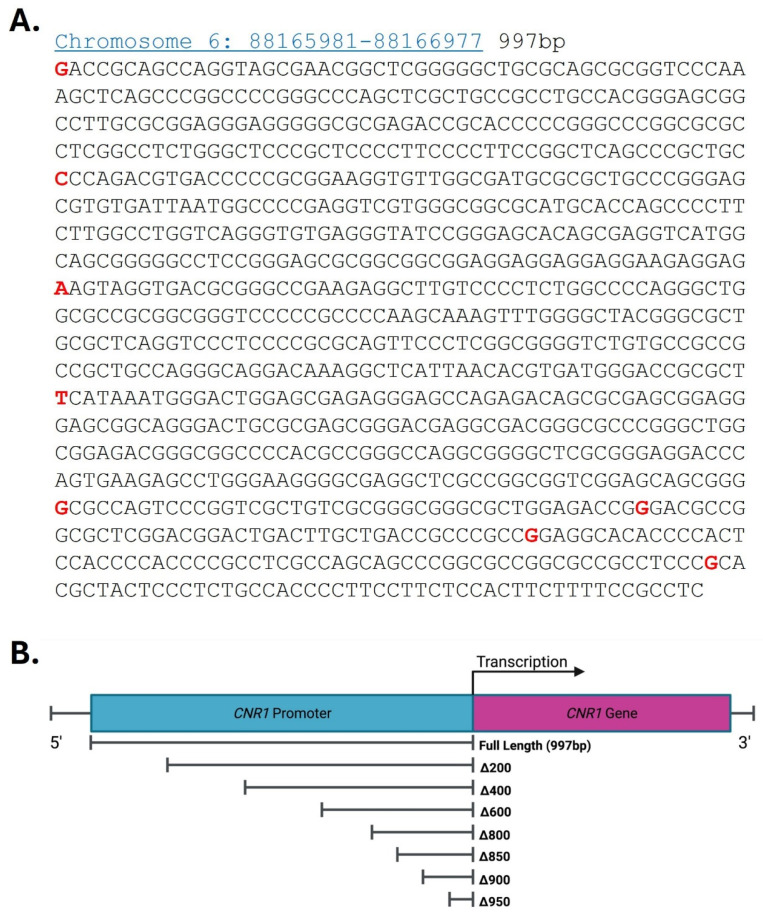
Graphical representation of the *CNR1* gene promoter sequence. (**A**) 997-base-pair promoter sequence from 88165981 to 88166977 on chromosome 6. A series of deletion fragments were generated starting from the 5′ end of the *CNR1* promoter and named according to the deleted base pairs (red letters mark the start of each deletion fragment). (**B**) Schematic depiction of the *CNR1* promoter, transcription start site (TSS), and the beginning of the *CNR1* gene sequence as well as the relative position of each promoter deletion construct. Created with BioRender.com.

**Figure 2:**
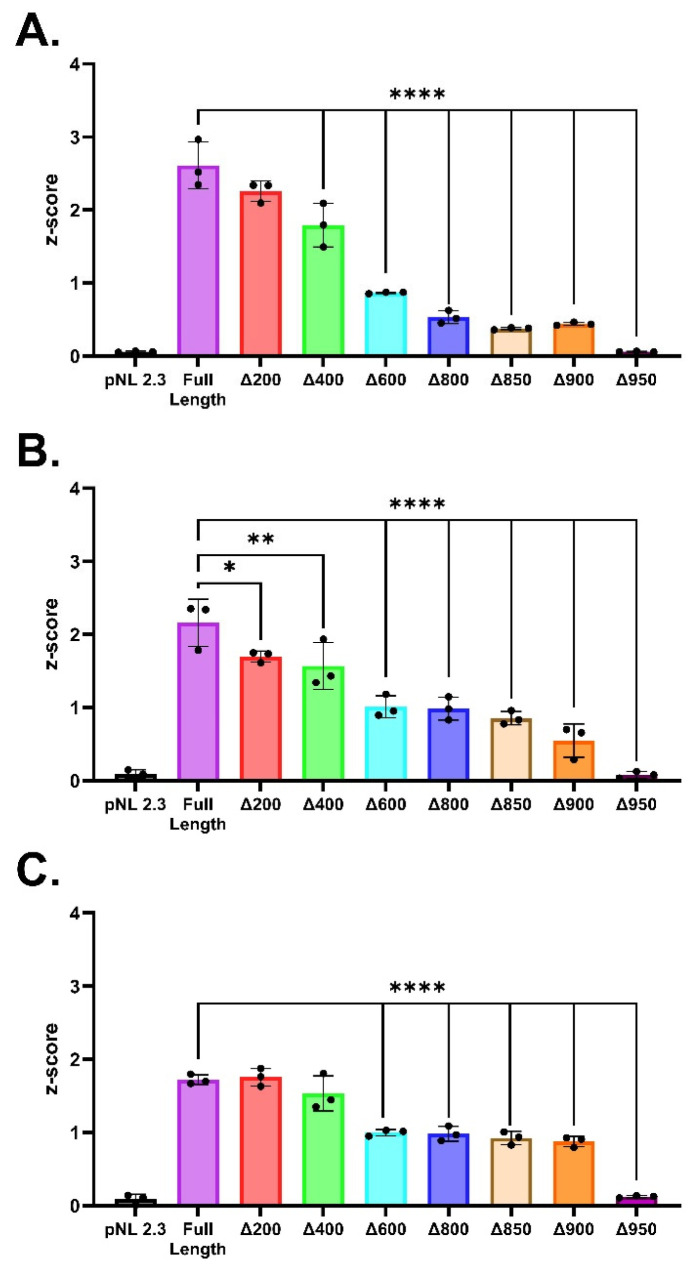
Luciferase reporter activity of full-length (FL) *CNR1* promoter and deletion fragments. The FL promoter and deletion fragments were transfected into HEK293T (**A**), HCT116 (**B**), and SHSY5Y (**C**) cells. Luciferase reporter activity was measured after 24 h, and the activity of each fragment was calculated as a z-score of the average relative light units (RLUs) from each experiment. All deletion fragments missing the first 400 bp (i.e., Δ600, Δ800, Δ850, Δ900, and Δ950) showed statistically significant reduction in activity in all three cell lines. In particular, the deletion of the regions between Δ400–Δ600 and Δ900–Δ950 marks significant reductions in activity in all three cell lines. Data shown are represented as z-score mean ± S.D. with individual replicates shown (*N* = 3). The statistical differences were evaluated by ordinary one-way ANOVA followed by Dunnett’s multiple comparison test, * *p* < 0.05, ** *p* < 0.01, **** *p* < 0.0001.

A 997-bp fragment immediately upstream of the reported *CNR1* transcription start site (TSS) (Full-Length, FL) and its deletion fragments (illustrated in Figure 1 and Figure S1) were cloned into a pNL2.3 luciferase reporter construct and transiently transfected into HEK293T (Figure 2A), HCT116 (Figure 2B) and SHSY5Y (Figure 2C) cells [1]. As noted in the Discussion section, these are commonly used human cell lines from tissues known to express the CB1R and where the receptor is thought to contribute to organ physiology and/or pathology. Deletion mapping of the promoter region revealed two major regulatory regions within the promoter, as assessed by a decrease in luciferase activity (Figure 2). The first major decrease occurred following the deletion of the first 600 bp, resulting in about a 50% reduction in luciferase activity across all three cell lines. Deletion of the region between Δ900 and Δ950 promoter fragments completely abolished luciferase activity (reducing it to empty vector levels). These data suggest that a key regulatory region is between the Δ400 and Δ600 fragments, and the core promoter sequences are between Δ900 and Δ950 fragments (within 100 bp of the reported transcription start site) [1].

### 2.2. Luciferase Activity of Full-Length CNR1 Promoter After Forskolin (FSK) Treatment

**Figure 3:**
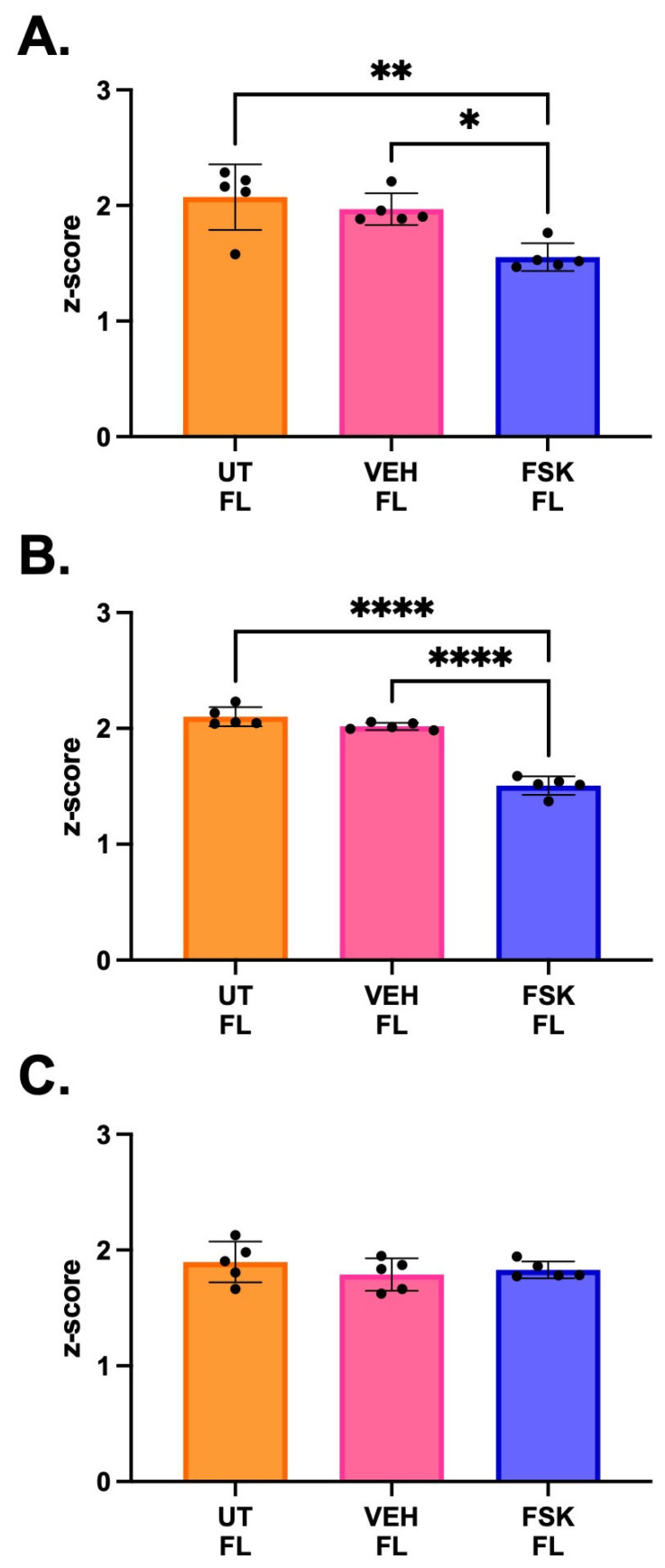
Luciferase reporter activity of full-length (FL) *CNR1* promoter after forskolin (FSK) treatment. The transfected FL promoter was treated with 5 µM FSK for 24 h, and the reporter activity was measured on untreated (UT), DMSO (VEH) and forskolin (FSK) samples. FSK treatment decreased the luciferase reporter activity in HEK293T (**A**) and HCT116 (**B**) cells. Meanwhile, FSK treatment in SHSY5Y (**C**) cells did not show the same reduction in reporter activity, demonstrating a subtle cell-specific response to increased levels of cAMP in the transcriptional regulation of *CNR1*. Data are represented as z-score mean ± S.D. with individual replicates shown (*N* = 5). The statistical differences were evaluated by ordinary one-way ANOVA followed by Tukey’s multiple comparison test, * *p* < 0.05, ** *p* < 0.01, **** *p* < 0.0001.

We next examined the possible regulation of transcription by GPCRs, (such as the G_i_/G_o_-coupled CB1R). To test this, we used forskolin (FSK) which activates adenylyl cyclase and increases cyclic AMP (cAMP) levels, a major downstream pathway component of GPCR activation. Increased cAMP levels within a cell ultimately activate CREB/CREM transcription factors. Cells transfected with the full-length *CNR1* promoter construct were treated with 5 μM FSK for 24 h (Figure 3). After FSK treatment, the luciferase reporter activity in HEK293T (Figure 3A) and HCT116 cells (Figure 3B) was significantly decreased. However, this decrease in reporter activity was less pronounced and not significant in SHSY5Y (Figure 3C). These results demonstrate a slight cell-type specific response to FSK and cAMP levels on transcriptional activation/inhibition of the *CNR1* promoter.

### 2.3. Luciferase Activity of Promoter Deletions After Forskolin (FSK) Treatment

**Figure 4:**
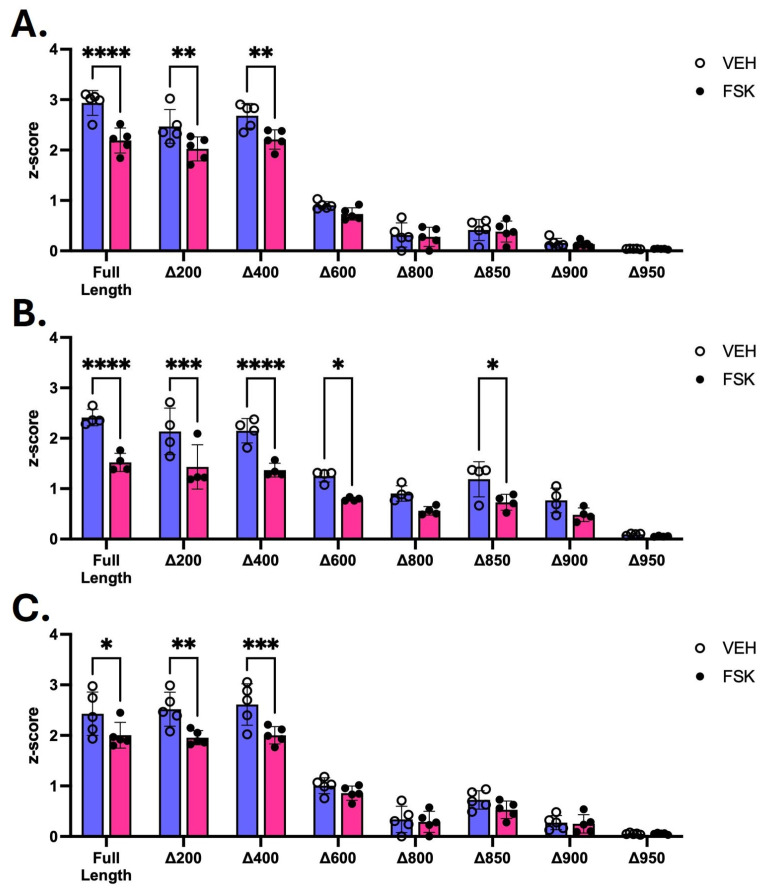
Luciferase reporter activity of full-length (FL) *CNR1* promoter and deletion fragments after forskolin (FSK) treatment. The FL promoter and deletion fragments were transfected and treated with 5 µM FSK for 24 h. Luciferase reporter activity assays were performed on both FL promoter and deletion fragments with VEH (DMSO) and 5 µM forskolin (FSK) treatment. The reporter activity of the FL promoter and each fragment was calculated as a z-score of the average relative light units (RLUs) from each experiment. All three cell lines (**A**–**C**) showed the previously described decrease in activity after deletion of the regions between Δ400–Δ600 and Δ900–Δ950. In HEK293T (**A**) and HCT116 (**B**) cells, full-length, Δ200, and Δ400 fragments showed reduction in transcriptional activity, as measured by luciferase activity following FSK treatment when compared to their untreated controls. The impact of FSK treatment appears reduced in SHSY5Y cells (**C**). Data are represented as z-score mean ± S.D. with the average of individual replicates shown (*N* = 5 (HEK293T and SHSY5Y); *N* = 4 (HCT116)). The statistical differences were evaluated by ordinary two-way ANOVA with Šídák’s multiple comparisons test, * *p* < 0.05, ** *p* < 0.01, *** *p* < 0.001, **** *p* < 0.0001.

Because of the observed cAMP-induced decreases in luciferase activity with the full-length construct, suggesting the presence of cAMP-responsive elements (CRE) within the promoter, we next mapped the location of potential elements using the promoter fragment constructs (Figure 4). Treatment of transfected HEK293T (Figure 4A) and HCT116 (Figure 4B) showed a decrease in promoter activity with the three largest constructs, but the effect of forskolin on luciferase activity was greatly reduced following deletion of the first 600 bp (Δ600 fragments and larger deletions). Interestingly, we also observed a smaller, but still significant decrease in cAMP responsiveness in SHSY5Y cells for these three larger promoter fragments (Figure 4C). These results should be considered with reservations as the transient transfection in SHSY5Y cells was less effective, and luciferase assay results showed a greater degree of variability. Furthermore, while the cAMP-induced reduction was less, we observed a continued impact of FSK treatment across the smaller promoter fragments in HCT116 cells. In sum, these data suggest the presence of a potential CRE within the major regulatory region—the 200 bp between the Δ400–Δ600 fragments—of the *CNR1* promoter. This suggests that this element(s) may be responsible for the decrease in expression observed in the deletion series described in Figure 2.

### 2.4. CREB/CREM Expression in HEK293T, HCT116, and SHSY5Y Cells

**Figure 5:**
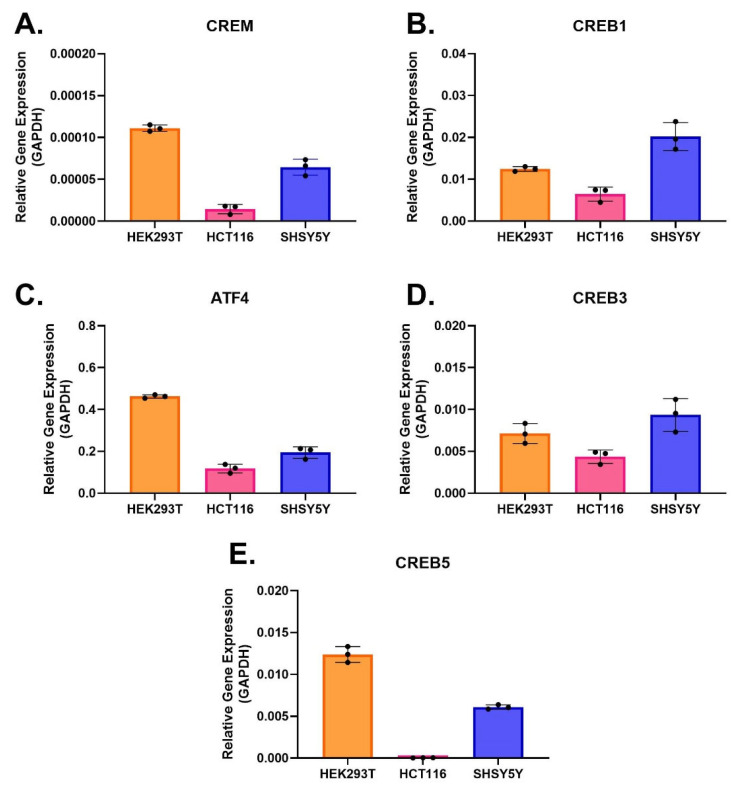
Endogenous expression of cAMP response elements (CREs)-related transcription factors in untreated HEK293T, HCT116 and SHSY5Y cells. The expression of CRE-related transcription factors, including cAMP-responsive element modulator (CREM; (**A**)), cAMP response element-binding proteins (CREB 1, 3 and 5; (**B**,**D**,**E**)), and activating transcription factor 4, (ATF4, previously known as CREB2; (**C**)), were measured in all three cell lines. Expression of transcription factors was compared to the expression of GAPDH. Data are represented as mean ± S.D. with individual replicates shown (*N* = 3). The statistical differences were evaluated by ordinary one-way ANOVA followed by Tukey’s multiple comparison test for each transcription factor (no significance).

Because of observed cAMP-induced responses to, and potential differences in cell lines to forskolin (FSK) treatment(Figure 3 and Figure 4), we examined the expression levels of CREB and CREM mRNA in each of these cell lines. We tested CREM (Figure 5A), CREB1 (Figure 5B), ATF4 (formerly CREB2; Figure 5C), CREB3 (Figure 5D), and CREB5 (Figure 5E) in all three cell lines. Expression of ATF4 (Figure 5C) was the highest for each of the three cell lines, while expression of CREM (Figure 5A) was the lowest in each cell line. Regardless, we did not observe any significant differences in CREB or CREM gene expression that could account for these differences; however, CREM and CREB5 expression are notably low in HCT116 cells.

### 2.5. CNR1 Gene Endogenous-Expression Regulation After Forskolin (FSK) Treatment

**Figure 6:**
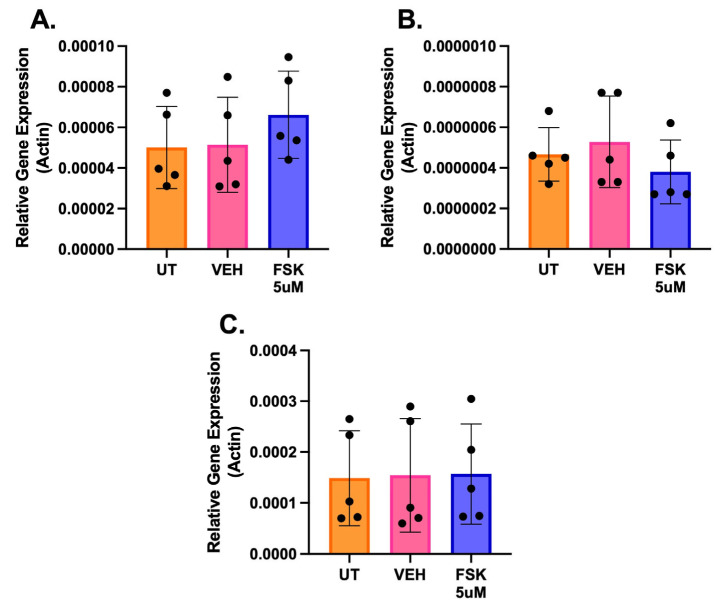
Endogenous expression of cannabinoid receptor type 1 (*CNR1*) gene after 24 h treatment with 5 µM forskolin (FSK) in HEK293T, HCT116 and SHSY5Y. All three cell lines were untreated (UT), treated with DMSO (VEH), or 5 µM forskolin (FSK) for 24 h. The expression of *CNR1* was measured and plotted against the relative expression of actin of HEK293T (**A**), HCT116 (**B**), or SHSY5Y (**C**). Data are represented as mean ± S.D. with individual replicates shown (*N* = 5). The statistical differences were evaluated by ordinary one-way ANOVA followed by Tukey’s multiple comparison test for each cell line (no significance).

We next tested the effect of forskolin (FSK) treatment on the endogenous expression of the *CNR1* gene. In contrast to what we observed with FSK treatment of the luciferase constructs, we found FSK treatment had no significant impact on the endogenous expression of the *CNR1* gene in HEK293T (Figure 6A), HCT116 (Figure 6B) and SHSY5Y (Figure 6C) cell lines. Not surprisingly, this observation suggests that additional regulatory elements, outside of the 997-bp promoter region, regulate *CNR1* expression.

### 2.6. In Silico Identification of Promoter Elements

**Figure 7:**
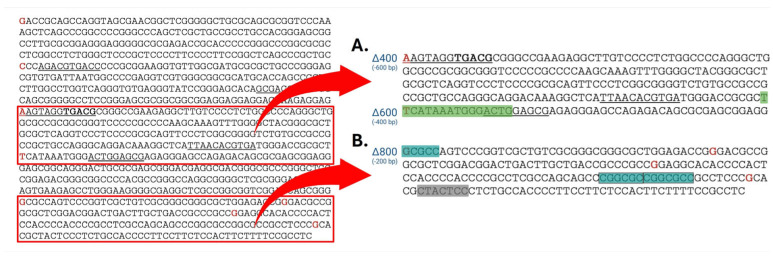
Location of potential cAMP response elements (CREs) and promoter elements in the full-length (FL) *CNR1* promoter sequence. In silico modeling of the FL promoter sequence predicted several CREs in two online software packages: JASPAR (underlined) predicted multiple binding sequences while **PROMO** (bold) only predicted one area of binding (red letters mark the start of each deletion fragment). (**A**) Both software packages predicted a CRE at the start of Δ400 (−600 bp from TSS) maintaining luciferase activity similar to FL. Deletion of this CRE in Δ600 (−400 bp from TSS) resulted in significantly reduced activity. In (**A**,**B**), the eukaryotic core promoter predictor YAPP found multiple promoter elements in the two regulatory regions, including TATA box (TATA, green), B recognition elements (BRE, turquoise), and initiator elements (Inr, gray). The regions between Δ900 (−100 bp from TSS) and Δ950 (−50 bp from TSS) showed core promoter function that, following deletion, completely eliminated transcriptional activity. Created with BioRender.com.

Two software packages for predicting transcription-factor binding profiles of CREB/CREM elements were utilized to identify potential regulatory elements within the key regulatory region between Δ400–Δ600 (−600 bp and −400 bp) of our luciferase promoter constructs (Figure 4). JASPAR predicted multiple binding sequences, while PROMO only predicted one area of binding; additionally, another potential CRE site was visually identified upon analysis of the sequence (Figure 7A). Of particular interest is a CRE site predicted by both PROMO and JASPAR located close to the start of the Δ400 fragment (located about −600 bp from the transcription start site), which is the last fragment to maintain a large luciferase promoter activity.

The online software package YAPP was used to identify potential core promoter elements found between the Δ900 and Δ950 fragments, which represented deletion of the core promoter region (Figure 2). This analysis predicted two overlapping BRE elements and an INR element within this region that may represent the core promoter elements of *CNR1* (Figure 7B).

### 2.7. Transcriptional Activity of BRE and Inr Mutations Within Full-Length CNR1 Promoter

**Figure 8:**
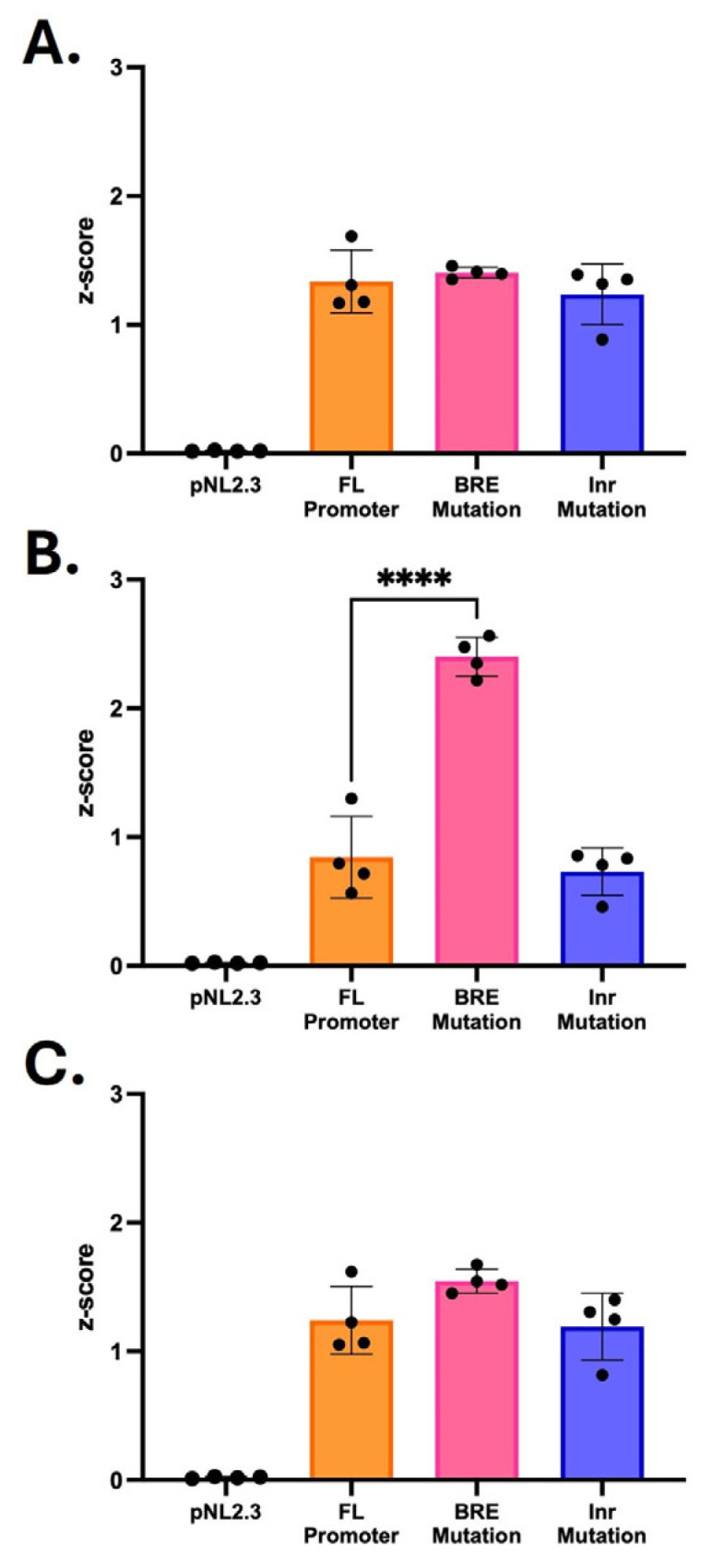
Luciferase reporter activity of BRE- and Inr-mutated full-length *CNR1* promoter. The full-length (FL) *CNR1* promoter was separately mutated at the BRE and Inr promoter elements. The unmutated FL promoter, BRE-mutated FL promoter, and Inr-mutated FL promoter were transfected into all three cells line. Luciferase reporter activity assays were performed, and all three FL promoters were tested. The reporter activity for each promoter was calculated as a z-score of the total relative light units (RLUs) from each experiment. HEK293T (**A**) and SHSY5Y (**C**) did not show significant changes in activity at either mutation. In HCT116 (**B**), the BRE-mutated FL promoter displayed a two-fold increase in luciferase activity when compared to unmutated FL. Data are represented as z-score mean ± S.D. with individual replicates shown (*N* = 4). The statistical differences were evaluated by ordinary one-way ANOVA followed by Dunnett’s multiple comparison test. **** *p* < 0.0001.

After identifying two different types of predicted promoter elements within the last ~100bp of the reported transcription start site (TSS), the proximal BRE and Inr were mutated within the full-length (FL) *CNR1* promoter sequence, as previously reported [13,14,15,16,17,18,19,20]. Specifically, the proposed BRE sequence of C**G**GC**G**CC was modified to C**A**GG**A**CC and the proposed Inr sequence of CT**A**C**T**CC was converted to CT**T**C**G**CC. Luciferase reporter assays were performed with all three cell lines to quantify the transcriptional activity of the FL containing each mutation (Figure 8). In HEK293T and SHSY5Y cells, the mutation of either BRE or Inr did not produce any significant changes in transcriptional activity when compared to unmutated FL *CNR1* promoter. However, in HCT116 cells, a two-fold increase in activity of the BRE mutated construct was observed when compared to unmutated *CNR1* promoter. Meanwhile, the Inr-mutated FL only showed a minimal reduction in activity without statistical significance.

## 3. Discussion

In this study, we mapped potential regulatory regions within the *CNR1* promoter. Our findings indicate that two major regulatory regions exist: one distal between Δ400 and Δ600 (−600 bp and −400 bp relative to the reported transcription start site), and a second more proximal region between Δ900 and Δ950 (−100 bp and −50 bp relative to the reported transcription start site) [1]. The deletion of the region between Δ400 and Δ600 reduced luciferase expression in all three cell lines by about 50%, while the deletion of the region between Δ900 and Δ950 abolished all remaining luciferase expression. These findings suggest that an active basal promoter region for *CNR1* lies within these 50 base pairs, and the 200 base pairs between Δ400 and Δ600 contain a major transcriptional regulatory region.

To evaluate the functional relevance of these regulatory regions across biologically distinct cellular contexts, promoter activity was examined in three commonly used human cell lines representing tissues in which CB1 receptor signaling is known to play important physiological or pathological roles [13,14,15,16,17,18,19]. HEK293T cells were selected as a broadly utilized human cell line that serves as a standard platform for molecular and transcriptional studies and is frequently used in cannabinoid receptor binding assays, generally over-expressing the receptor of interest [20,21,22]. Furthermore, CNR1 has been found to play a role in kidney disease, renal fibrosis and diabetic nephropathy. HCT116 colorectal cancer cells were included because alterations in CB1 receptor expression and endocannabinoid signaling have been reported in both inflammatory bowel disease (IBD) and colorectal cancer, suggesting a role for *CNR1* regulation in gastrointestinal health and disease. Previously, we confirmed the expression of CNR1 mRNA in both HEK293T and HCT116 cells [23]. SH-SY5Y neuroblastoma cells were selected as a neuronal model because CB1 is the most abundant GPCR in the brain and is widely expressed on neurons. CB1R has also been found to have protective roles in neurodegenerative disorders, and decreased *CNR1* mRNA is a known feature of Huntington’s disease [24,25].

To begin to identify pathways that may contribute to regulation of the CNR1 promoter, the impact of cAMP levels on transcription was tested. We found that HEK293T and HCT116 cell treatment with FSK produced a significant reduction in luciferase expression of the full-length construct; however, this effect was more modest in SHSY5Y cells, suggesting differential regulation by cAMP response element (CRE) transcription factors between the cell lines. Treatment of the deletion fragments with FSK decreased luciferase expression by varying degrees; of note, the FSK-mediated decrease was absent in constructs that deleted the first 600 bp, with some exceptions in HCT116 cells. This suggests that a regulatory CRE element resides within the 200 bp separating the Δ400 and Δ600 constructs (the region between −600 bp and −400 bp relative to the transcription start site). Consistent with this hypothesis, in silico analysis identified potential cAMP responsive elements (CRE) within the 200-bp region between Δ400–Δ600. To account for the cell-type differences in cAMP responsiveness, we examined the expression levels of CREB/CREM transcription factors across the three cell lines; however, no notable differences in CREB/CREM transcription factor expression were observed. This further suggests that other elements were mediating part of the reduced promoter activity with FSK treatment. Furthermore, endogenous expression of *CNR1* was not impacted by FSK treatment. This discrepancy between the promoter-reporter assays and the endogenous gene expression raises the possibility that additional regulatory elements outside the cloned promoter region or chromatin-dependent mechanisms not captured by the reporter constructs may contribute to the regulation of endogenous *CNR1* transcription.

Beyond the putative CRE, in silico mapping identified potential BRE and Inr promoter elements between Δ900 and Δ950 and within Δ950, respectively, that may account for the changes in expression we observed. The BRE (B recognition element) and Inr (initiator element) interact with transcription factor II B (TFIIB) and RNA polymerase II (PolII), respectively, and collaborate in direct transcription initiation [26,27,28]; while still consistent with a minimal promoter being present in this small region, this demonstrates that the suggested BRE and Inr may not play a key role in regulating transcription. Additionally, other elements may be present in the larger constructs that can drive transcription, and the identified BRE and INR may only be active in the smaller promoter fragment constructs. Further studies to refine the map of the CNR1 promoter, including the impact of mutations of the BRE and INR in a smaller construct, will provide additional, important insight into the regulation of *CNR1*.

Most of the information regarding *CNR1* gene regulation comes from genomic variation, transcription-factor binding, and associated single-nucleotide polymorphisms (SNPs) [1,4,8,9,10,12]. Zhang et al. investigated promoter/enhancer activity in sequences that extended 1.0, 2.0 and 3.0 kb 5′ from the human *CNR1* gene exon 1 transcription start site (TSS) [1]. This group reported higher luciferase expression from the 1.0 kb sequence, compared to the 2.0 and 3.0 kb fragments, suggesting distal inhibitory elements. Beyond the promoter region, the expression of genes can be influenced by enhancer/repressor sequences, the expression of transcriptional factors or co-factors, and/or by mutations in the DNA sequence (i.e., SNPs) [4,8,9,10,12]. Studies have identified potential crosstalk, co-expression, and co-localization of the CB1R with other receptors [2,3,29]. One study found that the testosterone-androgen receptor (AR) complex, known to function as a transcription factor, enhances the transcription of *CNR1* in SHSY-5Y cells transfected with AR and *rCNR1* [2,29]. The increase in expression of the *rCNR1* was due to two potential AR binding sites in the 1.5 kb rat *CNR1* promoter region. The location of these AR binding sites, within the first 500 and 900 bp upstream of the *rCNR1* TSS, coincides with the promoter region investigated in our study and may exert similar influences in the transcriptional regulation of the human *CNR1* gene. This is worth further study because AR has been reported to be a target for some phytocannabinoids and could suggest that phytocannabinoids’ inhibition of AR may impact *CNR1* expression [30,31].

A less considered factor that can lead to the transcriptional regulation of genes, including the *CNR1* gene, and can contribute to the development of other disorders is the occurrence of SNPs [1,8,9,10]. The SNP-related downregulation of rs806371, located 5′ from the coding *CNR1* exon 3, was studied as a variant associated with lower levels of high-density lipoprotein (HDL) cholesterol in humans [8]. Feng et al. concluded that this SNP acted as a novel regulatory binding element reducing the level of *CNR1* expression [8]. At the same time, rs2180619, along with related genotypes (GG), located 2.0 kb upstream of our promoter sequence, was shown to lower the efficiency of the endocannabinoid system due to lowered *CNR1* expression, producing a reduction in attention control and working memory as well as the increased risk of addiction and anxiety [1,9,11]. Lowered expression of *CNR1* is not the only way in which SNPs can lead to disorders. The rs1049353, identified in individuals with anorexia nervosa (AN), bulimia nervosa (BN), obesity, type 2 diabetes mellitus and major depression, is silent and does not alter the amino acid sequence, but it is thought to alter the mRNA stability [5,32,33,34].

Altered CB1R expression has been associated with numerous diseases and conditions. Increased CB1R expression has been reported in neuropathic pain, certain cancers (notably prostate cancer), cirrhosis, fatty liver disease, and depression [4,15,25,34,35,36,37,38,39,40,41,42,43,44]. Expression has been found to be decreased in Huntington’s disease, breast cancer, inflammatory bowel disease (IBD) and cannabis use disorder (CUD) [16,24,45,46,47,48,49]. In animal models of neuropathic pain, CB1R upregulation may reduce neurotransmitter release while enhancing responsiveness to exogenous cannabinoids such as THC [35,36,37]. Similarly, elevated CB1R expression in high-fat-diet-fed mice can be reversed by CB1R antagonists such as rimonabant [35,44]. In colorectal cancer there are conflicting data; loss of CB1R expression has been attributed to methylation of the *CNR1* promoter, whereas other studies have found higher levels of CB1R expression in patients with a poorer prognosis [15,50,51]. These observations highlight the importance of understanding the mechanisms regulating *CNR1* transcription. Changes in CB1 expression may also result from receptor internalization and degradation, processes implicated in cannabis tolerance [52,53]. Cannabis dependence and tolerance are associated with CB1R downregulation that correlates with the duration of chronic cannabis use and is largely reversible following prolonged abstinence [45,46]. Notably, reduced CNR1 mRNA expression is an early marker of Huntington’s disease [41].

Cannabis is widely used to manage symptoms associated with pain, depression, anxiety, and IBD, with many patients reporting therapeutic benefit [52,53,54]. At the same time, increasing use of high-THC cannabis products has raised concerns regarding adverse outcomes, including tolerance, CUD, cannabis-induced persistent psychosis, and cannabis-induced hyperemesis [55,56] A better understanding of the regulatory regions that modulate *CNR1* transcription may provide novel insights into the mechanisms contributing to these conditions and side effects.

The focus of the current study was to map regulatory regions within the promoter region of *CNR1*; however, a key limitation of this study is that it does not address other enhancer and insulator DNA elements. Two regulatory regions were identified; the first region showed a cAMP response element (CRE) and, after in silico analysis, the second region showed two overlapping BRE promoter elements and an Inr promoter element. Our data on the impact of FSK on endogenous *CNR1* expression, and other studies linking *CNR1* expression to cAMP levels, clearly indicate that these factors will be involved in regulating *CNR1* expression [3,57,58,59]. Mutation of the proximal BRE or Inr did not display core promoter functionality and, in the case of HCT116, it showed the opposite effect by resulting in a two-fold increase in expression only in the BRE-mutated FL promoter. Mapping of these additional elements will be key to better understanding how *CNR1* is potentially regulated and will provide additional insights into the observed cell-type-dependent differences observed here. Nonetheless, our results bring light to a new area in the transcriptional regulation of the *CNR1* expression, focusing on transcription factors and the activation of other GPCRs.

In conclusion, our results provide evidence for the presence of multiple regulatory elements within the 1000-bp sequence upstream of the *CNR1* transcriptional start site. Deletion analysis identified a proximal region located within the first 100 bp upstream of the transcriptional start site (between the comprised Δ900 and Δ950 fragments) that appears to be important for basal promoter activity and contains putative INR and BRE elements identified through in silico analyses. In addition, upstream we identified a potential regulatory region located between Δ400 and Δ600 that contains a putative cAMP response element (CRE). While the promoter-reporter studies are consistent with a role for this region in cAMP-responsive transcription, the absence of changes in the endogenous *CNR1* mRNA following forskolin treatment suggests that regulation of the endogenous gene is more complex and may involve additional regulatory elements, chromatin-dependent mechanisms, or distal enhancer regions not present in the construct tested in this study.

Taken together, these results expand the current understanding of the CNR1 promoter architecture and identify candidate regulatory regions that warrant further investigation. However, because the conclusions are based primarily on transient reporter assays and in silico predictions, additional studies will be required to confirm the functionality of the proposed regulatory elements and to define their contribution to endogenous *CNR1* expression in physiologically relevant cellular contexts. Notably, the function of the INR and BRE may be more relevant to the smaller fragments than the full-length construct, and this warrants further evaluation. Additional promoter elements may lie upstream in the larger constructs, and these should be analyzed in greater detail as well. As such, this work provides a framework for future studies aimed at more comprehensively characterizing the transcriptional regulation of *CNR1* and ultimately CB1 receptor expression.

## 4. Materials and Methods

### 4.1. DNA Cloning and Preparation of CNR1 Gene-Promoter Deletion Fragments

Genomic DNA was extracted from the human colorectal adenocarcinoma cell line, DLD-1, using the QIAmp DNA Mini Kit (Qiagen, Germantown, MD, USA) following the manufacturer’s protocol. A 997-bp region of *CNR1* 5′ flanking region was amplified utilizing HotStarTaq^®^ DNA Polymerase (Qiagen, Germantown, MD, USA) between 88165981 and 88166977 on chromosome 6 (Figure 1). The identity of the amplified fragment was confirmed by DNA sequencing (Figure 1A). The amplified genomic DNA of 997 base pairs were cloned into the pNL2.3 [secNluc Hygro] vector (Promega, Madison, WI, USA) using KpnI and BglII restriction enzymes (Thermo Fisher Scientific, Waltham, MA, USA) to create a secreted luciferase reporter gene construct. The reporter plasmid was purified from transformed *E. coli* using the QIAprep Spin Miniprep Kit (Qiagen, Germantown, MD, USA). A series of 5′ deletions of 200 bp (Δ200), 400 bp (Δ400), 600 bp (Δ600), 800 bp (Δ800), 850 bp (Δ850), 900 bp (Δ900) and 950 bp (Δ950) were generated using QuickChange Lightning Site-Directed Mutagenesis Kits (Agilent, Santa Clara, CA, USA). The primers utilized for the amplification, cloning, deletion, and sequencing of DNA are provided in Supplemental Table S1.

### 4.2. Cell Culture and Transient Transfection

The human embryonic kidney cell line, HEK293T, the human colon cancer cell line, HCT116, and the human neuroblastoma cell line, SHSY5Y, were cultured in DMEM (Corning, Manassas, VA, USA) supplemented with 10% fetal bovine serum (FBS) (Cytiva, Logan, UT, USA, and GeminiBio, Sacramento, CA, USA), and 1% Antibiotic-Antimycotic (Anti-Anti) (Gibco, Grand Island, NY, USA). Cells were seeded at a concentration of 100,000 cells per well in 24-well culture plates. Cells were incubated overnight for 12 to 16 h and then transfected with recombinant plasmids and Lipofectamine 2000 Reagent (Invitrogen, Carlsbad, CA, USA) following the manufacturer’s recommendations. Following 4–6 h of incubation, medium was replaced with DMEM (with 10% FBS and 1% Anti-Anti) and cultured for 24 h. Cells were treated with forskolin (Sigma-Aldrich, Saint Louis, MO, USA) where indicated (at 5 μM) and cultured for 24 h before performing the luciferase assay on culture medium.

### 4.3. Luciferase Reporter Assay

After transfection, cells were incubated for 24 h with or without forskolin treatment. The culture medium was collected and centrifuged at 9500× *g* for 5 min at 4 °C. For HEK293T cells, 10μL of cleared supernatant was added per well into a 96-well Lumitrac microplate (Greiner Bio-One, Frickenhausen, Germany). For HCT116 and SHSY5Y cells, 20μL of cleared supernatant were added per well. Full-length promoter and deletion fragment reporter activity were measured using a SpectraMax i3 imaging cytometer (Molecular Devises, San Jose, CA, USA) and the Nano-Glo^®^ Luciferase Assay System (Promega, Madison, WI, USA) according to the manufacturer’s instructions. Each reaction was carried out in duplicate, and the results of these technical replicates were averaged. Experiments were conducted independently, with multiple replicates. Luciferase reporter activity assay results were calculated as z-scores by dividing the average of duplicate reactions by the average luminescence for each well in the plate (luciferase assay run).

### 4.4. qRT-PCR for Gene Expression

Cells utilized for the expression of cAMP response element (CRE)-related transcription factors were cultured without treatment. Cells utilized for cannabinoid receptor type 1 (*CNR1*) gene expression were treated with FSK as described above. In both cases, RNA was isolated using a RNeasy^®^ Mini Kit (Qiagen, Hilden, Germany). cDNA was synthesized utilizing a High-Capacity cDNA Reverse Transcription Kit with RNase inhibitor (AppliedBiosystems, Vilnius, Lithuania) with 1 µg of isolated RNA. cDNA was diluted to 1:50 (transcription factors) and 1:5 (*CNR1*) for RT-PCR using a TaqMan^®^ Fast Advanced Master Mix (AppliedBiosystems, Vilnius, Lithuania) utilizing Human TaqMan probes for CREM (Assay ID: Hs01582003_g1), CREB1 (Assay ID: Hs00231713_m1), ATF4 (Assay ID: Hs00909569_g1), CREB3 (Assay ID: Hs00197255_m1), CREB5 (Assay ID: Hs00191719_m1), *CNR1* (Assay ID: Hs01038522_s1), Actin (Assay ID: Hs99999903_m1), and GAPDH (Assay ID: Hs02786624_g1) (AppliedBiosystems, ThermoFisher, Waltham, MA, USA). The resulting C_T_ values were analyzed to calculate the expression of CRE-related transcription factors and *CNR1* relative to GAPDH or β-actin (2^−ΔCt^) in each of the cell lines.

### 4.5. In Silico Promoter Analysis

The online software packages JASPR (https://jaspar.elixir.no/, version 11, accessed on 2 February 2025) and PROMO (https://bioinformaticaupf.crg.eu/2003/projectes03/2.2/promotor/ALGGEN%20-%20Algorismica%20i%20Genetica.Promotors.html, accessed on 2 February 2025) were used to search the DNA sequence of the region between the Δ400 and Δ600 bp fragments for human cAMP response elements. Core promoter elements were searched within the full 997-bp promoter fragment studied using YAPP (https://bioinformaticshome.com/db/tool/YAPP) accessed on 2 February 2025).

### 4.6. BRE and Inr Mutations and Luciferase Reporter Assay

Mutations of the predicted proximal BRE and Inr were generated using Q5 Site-Directed Mutagenesis Kit (New England BioLabs, Ipbswich, MA, USA) and the plasmids were isolated from transformed *E. coli* as previously described. The primers utilized for the amplification, cloning and sequencing of DNA are provided in Supplemental Table S2. Luciferase reporter assays were performed independently, with multiple replicates, as previously described.

### 4.7. Statistical Analysis

Statistical analysis was conducted using Prism Software (GraphPad, Boston, MA, USA, version 10.4.1). Ordinary one-way and two-way ANOVA with Dunnett’s/Tukey’s and Šidák’s multiple comparison test was used to evaluate luciferase data. Levels of endogenous *CNR1* were compared using one-way ANOVA with Tukey’s multiple comparison test. All data are presented as mean ± standard deviation.

## 5. Conclusions

In conclusion, our results show the existence of two regulatory regions in the 1000-bp sequence upstream of the *CNR1* gene transcription start site. The core promoter is located within the first 100 bp comprising Δ900 and Δ950 (between −100 bp and −5 bp upstream of the TSS), and an upstream regulatory region is located between Δ400 and Δ600 (−600 bp and −400 bp upstream of the TSS) that contains a cAMP response element (CRE). These results add to the current knowledge of the regulation of *CNR1*/CB1R expression and provide insights into key regulatory regions of the promoter.

## Data Availability

All data are shown in the manuscript, and raw data is available at ScholarSphere.

## Supplementary Materials

The following supporting information can be downloaded at: https://www.mdpi.com/article/10.3390/molecules31132387/s1. Table S1. *CNR1* Promoter Region and Deletion Fragment PCR Primers. Table S2. *CNR1* promoter BRE and Inr Mutation PCR primers. Figure S1. Promoter Constructs and Identified CNR1 Transcripts.

## Abbreviations

The following abbreviations are used in this manuscript:

CB1R	Cannabinoid receptor type 1
CNR1	Cannabinoid receptor type 1 gene
cAMP	Cyclic adenosine monophosphate
CRE	cAMP response element
BRE	B recognition element
Inr	Initiator element
AEA	N-arachidonoylethanolamine
2-AG	2-arachidonoylglycerol
Δ^9^-THC	Δ^9^-tetrahydrocannabinol
CNS	Central nervous system
CUD	Cannabis use disorder
SNP	Single nucleotide polymorphism
CREM	cAMP responsive element modulator
CREB	cAMP-response element binding protein
ATF4	Activating transcription factor 4
TSS	Transcription start site
RLUs	Relative light units
GPCRs	G protein-coupled receptors
UT	Untreated
VEH	Vehicle
FL	Full-length promoter
