# Supplementary material for: Transcriptional Mapping of the Human Cannabinoid Receptor 1 (CNR1) Gene Promoter
Source: Molecules. 2026 Jul 7;31(13):2387. doi: 10.3390/molecules31132387 (PMC13363282; doi:10.3390/molecules31132387)
Supplement: Supplementary file 1 [file molecules-31-02387-s001.zip › molecules-4346565-supplementary.pdf]

Supplemental Table S1. *CNR1* promoter region and deletion fragment PCR primers.

Name	Sequences
***CNR1*-Forward**	5′-GACCGCAGCCAGGTAGCGAA-3′
***CNR1*-Reverse**	5′-GAGGCGGAAAAGAAGTGGAGAAG-3′
***CNR1*-Cloning-Kpnl-Forward**	5′-AAGCGACTGGAGGTACCGACCGCAGCCAGGTAGCGAA-3′
***CNR1*-Cloning-BaglII-Reverse**	5′-GCAACAGATCTGAGGCGGAAAAGAAGTGGAGAAG-3′
***CNR1*-800-Forward**	5′-GAACATTTCTCTGGCCTAACTGGCCGGTACCCCCAGACGTGACCCCCGCGGAAGGTGTTG-3′
***CNR1*-800-Reverse**	5′-CAACACCTTCCGCGGGGGTCACGTCTGGGGGTACCGGCCAGTTAGGCCAGAGAAATGTTC-3′
***CNR1*-600-Forward**	5′-GAACATTTCTCTGGCCTAACTGGCCGGTACCAAGTAGGTGACGCGGGCCGAAGAGGCTTG-3′
***CNR1*-600-Reverse**	5′-CAAGCCTCTTCGGCCCGCGTCACCTACTTGGTACCGGCCAGTTAGGCCAGAGAAATGTTC-3′
***CNR1*-400-Forward**	5′-GAACATTTCTCTGGCCTAACTGGCCGGTACCTCATAAATGGGACTGGAGCGAGAGGGAGC-3′
***CNR1*-400-Reverse**	5′-GCTCCCTCTCGCTCCAGTCCCATTTATGAGGTACCGGCCAGTTAGGCCAGAGAAATGTTC-3′
***CNR1*-200-Forward**	5′-GAACATTTCTCTGGCCTAACTGGCCGGTACCGCGCCAGTCCCGGTCGCTGTCGCGGGCGG-3′
***CNR1*-200-Reverse**	5′-CCGCCCGCGACAGCGACCGGGACTGGCGCGGTACCGGCCAGTTAGGCCAGAGAAATGTTC-3′

***CNR1*-150-Forward**	5′-CATTTCTCTGGCCTAACTGGCCGGTACCCCGGCGCTCGGACGGACTGACTTGCTG-3′
***CNR1*-150-Reverse**	5′-CAGCAAGTCAGTCCGTCCGAGCGCCGGGGTACCGGCCAGTTAGGCCAGAGAAATG-3′
***CNR1*-100-Forward**	5′-CATTTCTCTGGCCTAACTGGCCGGTACCACTCCACCCCACCCCGCCTCGCCAG-3′
***CNR1*-100-Revers**	5′-CTGGCGAGGCGGGGTGGGGTGGAGTGGTACCGGCCAGTTAGGCCAGAGAAATG-3′
***CNR1*-50-Forward**	5′-CATTTCTCTGGCCTAACTGGCCGGTACCGCACGCTACTCCCTCTGCCACCCCTTC-3′
***CNR1*-50-Reverse**	5′-GAAGGGGTGGCAGAGGGAGTAGCGTGCGGTACCGGCCAGTTAGGCCAGAGAAATG-3′
***CNR1*-Reading-Forward**	5′-GGCAGGACAAAGGCTCATTA′3′
***CNR1*-Reading-Reverse**	5′-TCACCTACTTCTCCTCTTCCTC-3′
**pNL 2.3-Reading-Forward**	5′-CTAGCAAAATAGGCTGTCC-3′
**pNL 2.3-Reading-Reverse**	5′-GCTCCGGTCTAGAACTATAGGAG-3′

Supplemental Table S2. *CNR1* promoter BRE and Inr Mutation PCR primers.

Name	Sequences
***CNR1*_BRE Forward**	5′-CAGCAGCCCGGCGCCAGCACCGCCTCCCGCACGC-3′
***CNR1*_BRE Reverse**	5′-GCGTGCGGGAGGCGGTGCTGGCGCCGGGCTGCTG-3′
***CNR1*_INR Forward**	5′-CGCCTCCCGCACGCTTCGCCCTCTGCCACCCCT-3′
***CNR1*_INR Reverse**	5′-AGGGGTGGCAGAGGGCGAAGCGTGCGGGAGGCG-3′

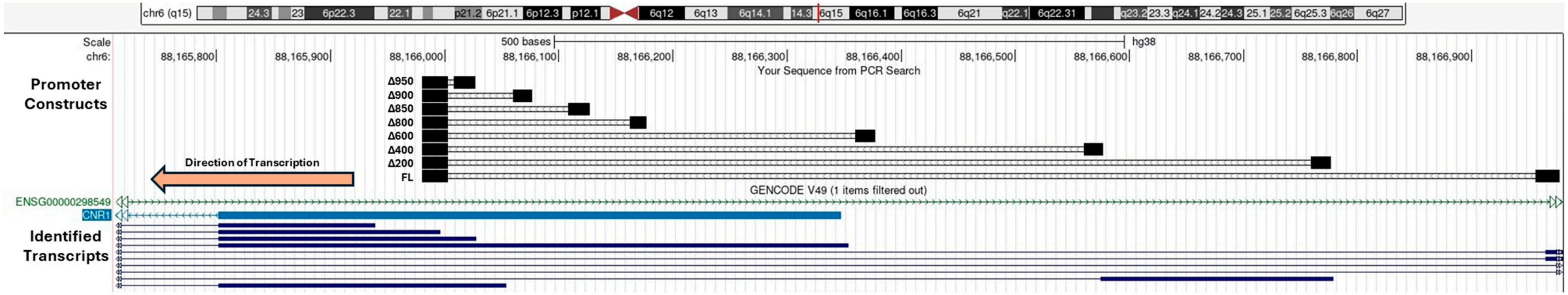

Supplemental Figure S1: Promoter Constructs and Identified CNR1 Transcripts. The top portion of this figure shows the promoter constructs used in this study (thick black boxes represent the primers). The bottom portion of the figure shows the mRNAs that have been identified from transcription of the CNR1 gene. Of note is that the CNR1 gene is located on the anti-sense strand. Figure was generated in UCSC Genome Browser on 10 June 2026.
